# Medical Schools of Louisville, Medical Education, Etc.

**Published:** 1884-04-20

**Authors:** 


### *MEDICAL SCHOOLS OF LOUISVILLE, MEDICAL EDUCATION, ETC.*

A somewhat bitter and vituperative quarrel has, for some time, been going on between the Louisville Medical College and the University of Louisville. Dr. D. W. Yandell, of the latter school, charges the former school with bad faith in respect to her obligations to other schools, and disreputable methods in obtaining pupils. The Louisville Medical College retorts with like charges against the University, as of persuading students from the Louisville Medical College by "unworthy arts and misrepresentations," etc.

It is certainly to be regretted that such quarrels extst between institutions of high position. We know that when a party is assaulted a reply is expected, and that a conflict commenced is not likely to terminate without engendering bad feeling and animosities that many years will not efface. But it is mortifying to the profession that snch feuds are sprung, and the more so that there seems to be cause for it. Where the chief fault is to be found, or whether or not both are involved, the public will probably soon ascertain, as the parties to the conflict will freely ventilate the facts in the case. At all events' the effect must be to lower the dignity and weaken the influence which the Louisville schools have heretofore maintained.

There is reason to fear that the competition between schools has led to serious abuses on the part of many institutions North and South. The honest schools—those which are seeking to elevate and ennoble the standard of the Profession—are placed at a great disadvantage by the unfairness and unprofessional methods of their competitors; and so little is understood of these things by the Profession and the public, and so little discrimination is made by students as between the true and the false, that great discouragement prevails among those who desire to remove the unprofessional methods which are practiced by competing schools.

The fact that no impartial arbiter exists, to whom such controversies may be referred, is unfortunate. Our state societies are too often run by cliques and rings to be relied upon to adjudicate such matters. The American Medical Association would be the better tribunal for such cases, and no higher or more important movement for reform could be instituted by the Profession than to invest the American Medical Association with such degree of authority and supervisory action, in respect to medical schools, as would correct the evils which, like a cancerous sore, are slowly but surely lowering the standard of education, corrupting the honor, and seriously striking at the very vitals of the Profession.

In certain of the European Governments the strong aim of the law removes all these difficulties by endowing the schools, requiring a preliminary examination and stipulating the conditions upon which diplomas are to be granted. Were our schools limited in number to one in a State, and the professorships endowed, thus removing all pecuniary incentive to secure large numbers of matriculants, and all students were required to go before an independent and salaried State Board of Examination, the evils now complained of could not exist, and the Profession would hold a position of honor, worth and dignity pre-eminent to all other callings in life. W.

